# The expression of PKM1 and PKM2 in developing, benign, and cancerous prostatic tissues

**DOI:** 10.3389/fonc.2024.1392085

**Published:** 2024-04-12

**Authors:** Lin Li, Siyuan Cheng, Yunshin Yeh, Yingli Shi, Nikayla Henderson, David Price, Xin Gu, Xiuping Yu

**Affiliations:** ^1^ Department of Biochemistry and Molecular Biology, LSU Health Sciences Center at Shreveport, Shreveport, LA, United States; ^2^ Feist-Weiller Cancer Center, LSU Health Sciences Center at Shreveport, Shreveport, LA, United States; ^3^ Pathology & Laboratory Medicine Service, Overton Brooks VA Medical Center, Shreveport, LA, United States; ^4^ Department of Urology, LSU Health Sciences Center at Shreveport, Shreveport, LA, United States; ^5^ Department of Pathology, LSU Health Sciences Center at Shreveport, Shreveport, LA, United States

**Keywords:** PKM1, PKM2, neuroendocrine, prostate cancer, metabolism

## Abstract

**Background:**

Neuroendocrine prostate cancer (NEPCa) is the most aggressive type of prostate cancer (PCa). However, energy metabolism, one of the hallmarks of cancer, in NEPCa has not been well studied. Pyruvate kinase M (PKM), which catalyzes the final step of glycolysis, has two main splicing isoforms, PKM1 and PKM2. The expression pattern of PKM1 and PKM2 in NEPCa remains unknown.

**Methods:**

In this study, we used immunohistochemistry, immunofluorescence staining, and bioinformatics analysis to examine the expression of PKM1 and PKM2 in mouse and human prostatic tissues.

**Results:**

We found that PKM2 was the predominant isoform expressed throughout prostate development and PCa progression, with slightly reduced expression in murine NEPCa. PKM1 was mostly expressed in stromal cells but low-level PKM1 was also detected in prostate basal epithelial cells. Its expression was absent in the majority of prostate adenocarcinoma (AdPCa) specimens but present in a subset of NEPCa. Additionally, we evaluated the mRNA levels of ten PKM isoforms that express exon 9 (PKM1-like) or exon 10 (PKM2-like). Some of these isoforms showed notable expression levels in PCa cell lines and human PCa specimens.

**Discussion:**

Our study characterized the expression pattern of PKM1 and PKM2 in prostatic tissues including developing, benign, and cancerous prostate. These findings lay the groundwork for understanding the metabolic changes in different PCa subtypes.

## 1 Introduction

Human prostate is a male reproductive accessory gland. It comprises multiple cell types, including luminal epithelial, basal epithelial, and stromal cells (1). Prostate cancer (PCa), the most prevalent cancer among American men, typically originates from luminal epithelial cells, leading to prostate adenocarcinoma (AdPCa) (2, 3). Androgen deprivation therapy (ADT) is the primary treatment for advanced AdPCa (4). However, ADT eventually fails, resulting in tumor progression to castrate-resistant PCa (CRPCa) (4). Once PCa becomes castrate-resistant, it often adopts an aggressive neuroendocrine (NE) phenotype, associated with high morbidity and an average survival of less than 1.5 years (5, 6).

Neuroendocrine prostate cancer (NEPCa) represents a highly aggressive subtype of PCa, accounting for approximately 30% of CRPCa cases (5, 6). While NE cells constitute only a minor subpopulation in the prostate epithelia and primary NEPCa is rare, NE differentiation commonly arises in PCa after prolonged androgen deprivation (5, 6). PCa with prominent NE differentiation is typically castrate-resistant and highly aggressive.

Genetically engineered mice are frequently used in PCa research. The transgenic adenocarcinoma mouse prostate (TRAMP) model is a common choice for NEPCa studies. TRAMP mice express SV40 T antigen in the prostate. They develop prostatic intraepithelial neoplasia (PIN) and, following castration, NEPCa (7–10).

The prostate displays a unique energy metabolism. In normal prostate luminal epithelial cells, there is a notable increase in glucose consumption compared to epithelial cells from other anatomical sites. This heightened glucose uptake is primarily directed towards citrate production for secretion (11). This process is facilitated by the accumulation of zinc within luminal epithelial cells, which acts as an inhibitor of m-aconitase, the enzyme responsible for converting citrate to isocitrate (12, 13). As a result of reduced m-aconitase activity, citrate oxidation is minimized, leading to citrate secretion and a truncated tricarboxylic acid (TCA) cycle (14).

Metabolism undergoes significant changes during the transformation of prostate epithelial cells (15–17). This transformation is marked by a reduction in intracellular zinc levels, leading to the derepression of m-aconitase function (15–17). Consequently, there is a shift in metabolism towards citrate oxidation, allowing the TCA cycle to proceed (11, 16). This alteration results in an increased TCA cycle flux in early-stage AdPCa cells compared to normal prostate epithelial cells (18). Consequently, the Warburg effect is relatively modest in early-stage PCa (14). However, as AdPCa progresses, including in advanced tumors, a more pronounced Warburg effect is observed (14, 18). This metabolic shift allows tumor cells to sustain high levels of glycolytic intermediates, supporting the biosynthesis required for their rapid proliferation (19, 20).

Regarding glucose metabolism in NEPCa, it remains largely unknown.

A pivotal enzyme governing glucose metabolism in cancer cells is pyruvate kinase muscle isozyme (PKM) (21, 22). PKM catalyzes the conversion of phosphoenolpyruvate to pyruvate, marking one of the three irreversible and heavily regulated steps in glycolysis (21, 22). PKM exists in two isoforms, PKM1 and PKM2, due to alternative splicing (21, 23). PKM1 includes exon 9 but not exon 10, whereas PKM2 includes exon 10 but not exon 9 (24). PKM1 is predominantly expressed in energy-consuming tissues such as the heart, muscle, and brain, where it forms a constitutively active tetramer promoting oxidative phosphorylation (25, 26). In contrast, PKM2 expression is more prevalent in embryonic tissues and cancer cells (27–29). Unlike PKM1, PKM2 can exist as a catalytically active tetramer or a less active dimer/monomer, with its stoichiometry regulated by allosteric factors and post-translational modifications (22, 30, 31). Similar to many other cancer types, advanced AdPCa exhibits high expression of PKM2, which predominantly forms less-active dimer (22).

In addition to PKM1 and PKM2, pyruvate kinase has two other isoforms, PKL and PKR, encoded by the PKLR gene under the control of different promoters (32, 33). PKL is primarily expressed in the kidney, liver, and intestine, while PKR is predominantly expressed in red blood cells (32).

In a recent study, using immunohistochemistry (IHC) staining, researchers evaluated the expression of PKM1 and PKM2 in prostatic tissues. Results from this study indicate that PKM1 is predominantly expressed in prostatic stromal cells, while PKM2 is expressed in both normal epithelia and cancer cells (34). Furthermore, increased expression of PKM2 in AdPCa compared to normal luminal epithelial cells was noted (35, 36). However, the expression of PKM1 and PKM2 in NEPCa remains poorly characterized.

In this present study, we aim to fill this gap by employing a combination of molecular biology techniques, including IHC and immunofluorescence (IF) staining, alongside bioinformatics-based RNA isoform quantification. We intend to assess the expression patterns of PKM1 and PKM2 during prostate development, in mouse models of PCa, and in human prostatic specimens, including benign human prostate (BPH), AdPCa, and NEPCa.

## 2 Materials and methods

### 2.1 Sample collection

Cell-derived xenograft tumors and prostatic tissues derived from wild type and TRAMP mice were from our archival collection. De-identified human tissues were obtained from LSU Health-Shreveport Biorepository Core, Overton Brooks VA Medical Center and Tissue for Research organization. The tissues include BPH, low-grade AdPCa, high-grade AdPCa, NEPCa, and NE tumors arising from pancreas, skin, lung, and bladder. Most tissues were from surgical resection except for five NEPCa cases that were from biopsies. All experiments were carried out in accordance with approved protocols by the Institutional Animal Care and Use Committee and Institutional Review Board of LSU Health-Shreveport.

### 2.2 IHC and IF staining

IHC staining was performed using Vectastain elite ABC peroxidase kit (Vector Laboratories, Burlingame, CA) as described previously (37). Primary antibodies include PKM1 and PKM2 (7067S, dilution 1:500, and 4053S, dilution 1:1000, respectively, Cell Signaling Technology, Danvers, MA), P63 and FOXA2 (ab735 and ab108422, respectively, dilution 1:500, Abcam, Cambridge, MA), HOXB13 and chromogranin A (CHGA) (sc-28333 and sc-1488, respectively, dilution 1:200, Santa Cruz Biotechnology, Dallas, TX), FOXA1 (A15278, dilution 1:500, Abclonal, Woburn, MA), Synaptophysin (SYP) (611880, dilution 1:1000, BD biosciences, San Jose, CA), and NKX3.1 (0314, dilution 1:500, Athena Enzyme Systems, Baltimore, MD). Images were taken using a Zeiss microscope (White Plains, NY). The intensity score was evaluated by a semiquantitative blinded manner and graded as 0 (negative), 1^+^ (low), 2^+^ (moderate), and 3^+^ (high). For IF staining, primary antibodies include CHGA (AB_1553436, dilution 1:50, Developmental Studies Hybridoma Bank, Iowa City, IA), PKM1, PKM2, P63, and SYP (source was the same as mentioned above, dilution 1:100). The IF staining was imaged with a Nikon fluorescence microscope (Melville, NY) as reported previously.

### 2.3 Cell culture

PCa cell lines (VCap, LNCaP, C4-2B, 22RV1, PC3, and DU145) were obtained from ATCC. These cells were cultured in RPMI 1640 supplemented with 10% FBS, and 1% penicillin-streptomycin. H660 cells were cultured in Prostalife media (LifeLine Cell Technology, Oceanside, CA). C2C12 cells were cultured in DMEM supplemented with 10% FBS, and 1% penicillin-streptomycin. Cells were maintained in an incubator at 37°C and 5% CO2.

### 2.4 Western blot and cross-linking

Cells were lysed in Laemmli SDS sample buffer followed by SDS-PAGE and Western blotting. Primary antibodies are beta-actin (sc-47778, dilution 1:500, Santa Cruz Biotechnology, Dallas, TX), PKM1 and PKM2 (the same source as mentioned above, dilution 1:1000), and HRP-conjugated secondary antibody (Cell Signaling, Beverly, MA). Protein bands were visualized by using ProSignal^®^ Dura ECL Reagent (Genesee Scientific, San Diego, CA) and Chemidoc™ Touch Imaging System (Bio-Rad). For the cross-linking experiments, cells were washed with ice-cold PBS three times and treated with 5mM disuccinimidyl suberate (DSS, A39267, Thermo Scientific, Waltham, MA) for 30 min at room temperature. The cross-linking reaction was stopped by adding the quenching solution (1M Tris, PH 7.5) to the final concentration of 20mM for 15 min. Then, cell lysates were used for WB as described above.

### 2.5 Bioinformatics analysis

RNAseq data collected in the CTPC dataset were analyzed to assess the expression of PKM isoforms using STAR-Salmon based transcript level quantification method (38–41). The raw Fastq files were downloaded from NCBI SRA database and dbGaP (phs000909.v1.p1 (42), phs000915.v2.p2 (43)). The raw count and TPM data of TCGA data were acquired from GDC Data Portal. NCBI human GRCh38 (release 106) and associated GFT files were acquired through “AWS iGenomes” (https://ewels.github.io/AWS-iGenomes/) and were used for alignment. FastQC (https://github.com/s-andrews/FastQC), Trim Galore (https://github.com/FelixKrueger/TrimGalore), STAR (38), Salmon (39) software were used for sequencing quality control, alignment and quantification in the Linux Ubuntu environment. The transcript expression was visualized in R (V4.3.0) environment. The TPM values were first log2 transformed and quantile normalized using “preprocessCore” R package (44). The data was visualized by boxplot using “ggplot2” R package (45).

Based on the inclusion of exon 9 or exon 10, the transcripts were classified into “PKM1-like” and “PKM2-like” isoforms, respectively. Among the PKM1-like transcripts, NM_182470.2 and NM_182471.2 transcripts encode the same protein since the only difference in them lies within their 5’ UTRs. The protein encoded by these two isoforms is known as PKM1. NM_002654.4 transcript encodes a protein that is known as PKM2. To coordinate our bioinformatics analysis of the ten PKM isoforms with the well-known PKM1 and PKM2, we summed the normalized log2-transformed TPM values of NM_182470.2 and NM_182471.2 as PKM1 transcript. The expression data of NM_002654.4 was directly used to reflect the levels of PKM2 transcript. Additionally, to complement our bioinformatics analysis with the PKM1/2 antibodies’ staining results, we summed the values of all exon 9-retained transcripts as PKM1-like expression data and all exon 10-retained transcripts as PKM2-like expression data.

### 2.6 Statistical analyses

The Chi-Square test was used to compare the protein expression of PKM isoforms between AdPCa and NEPCa of human PCa and student t-test was used to compare the levels of PKM transcripts between these groups.

## 3 Results

### 3.1 The expression of PKM1 and PKM2 in murine prostate

**Figure 1:**
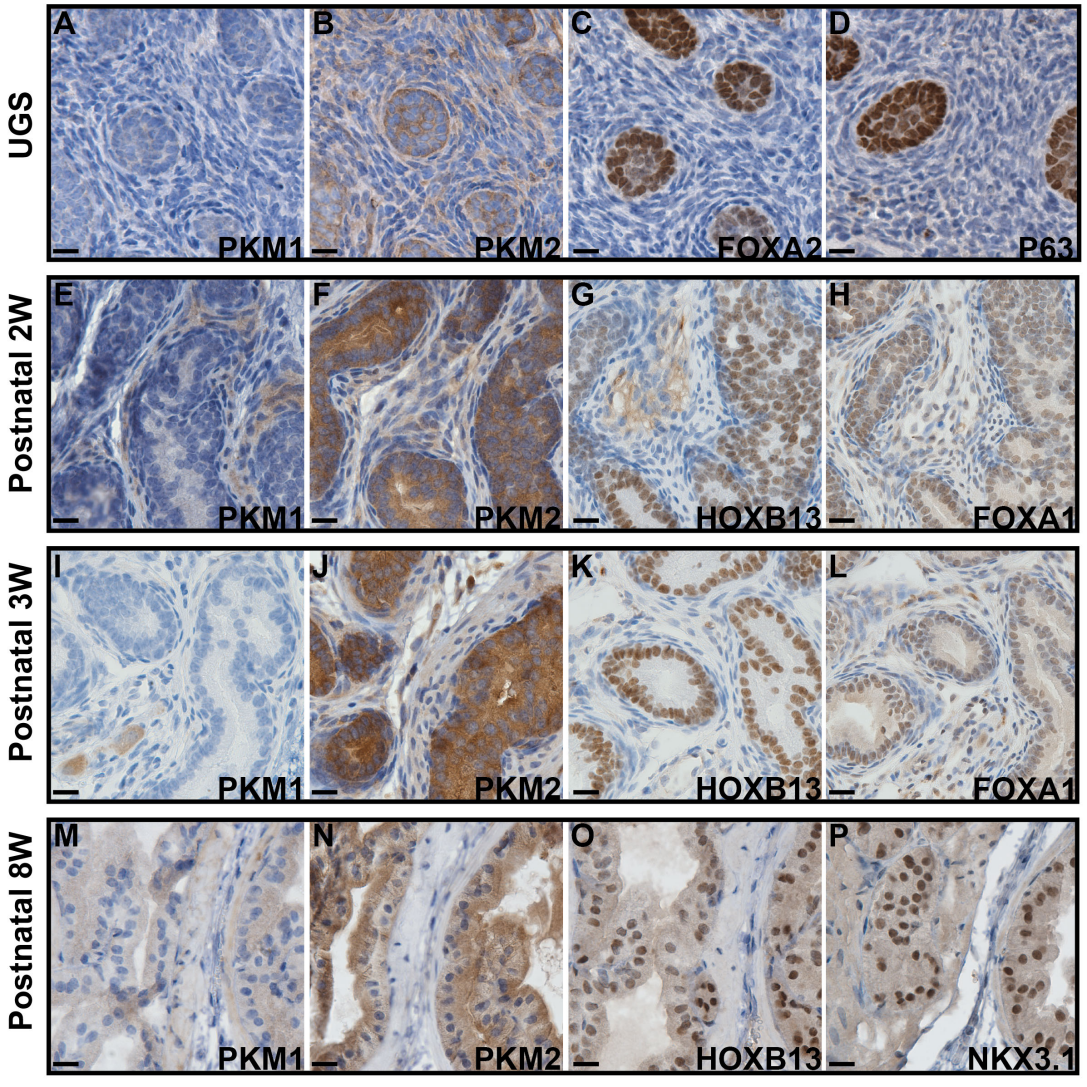
IHC staining to assess the expression of PKM1 and PKM2 in murine prostates at different developmental stages. **(A–D)** Urogenital sinus (UGS). **(E–H)** Postnatal 2 weeks prostate. **(I–L)** Postnatal 3 weeks prostate. **(M–P)** Postnatal 8 weeks prostate. The expression of FOXA2, P63, FOXA1, HOXB13, and NKX3.1 highlights the embryonic prostate buds and postnatal prostate luminal epithelial cells. Scale bar = 20 μM.

Using IHC, we examined the expression of PKM1 and PKM2 across murine prostate developmental stages ranging from the embryonic prostate undergoing budding morphogenesis (urogenital sinus, UGS), to the postnatal 2- and 3-week prostate, and the fully developed 8-week prostate. Our analysis revealed distinct expression patterns of both proteins. Specifically, we observed that PKM1 expression was largely absent during prostate development (including embryonic and postnatal 2–3-week prostate, **Figures 1A, E, I**) but minimal expression was detected in the fully developed 8-week prostate (**Figure 1M**). On serial sections, the embryonic prostate buds were highlighted by positive expression of FOXA2 and P63 (**Figures 1C, D**), and prostate luminal epithelial cells were marked by positive staining of HOXB13, FOXA1, and NKX3.1 (**Figures 1G, H, K, L, O, P**). In contrast, robust PKM2 expression was consistently detected in prostate epithelial cells throughout all developmental stages (**Figures 1B, F, J, N**).

**Figure 2:**
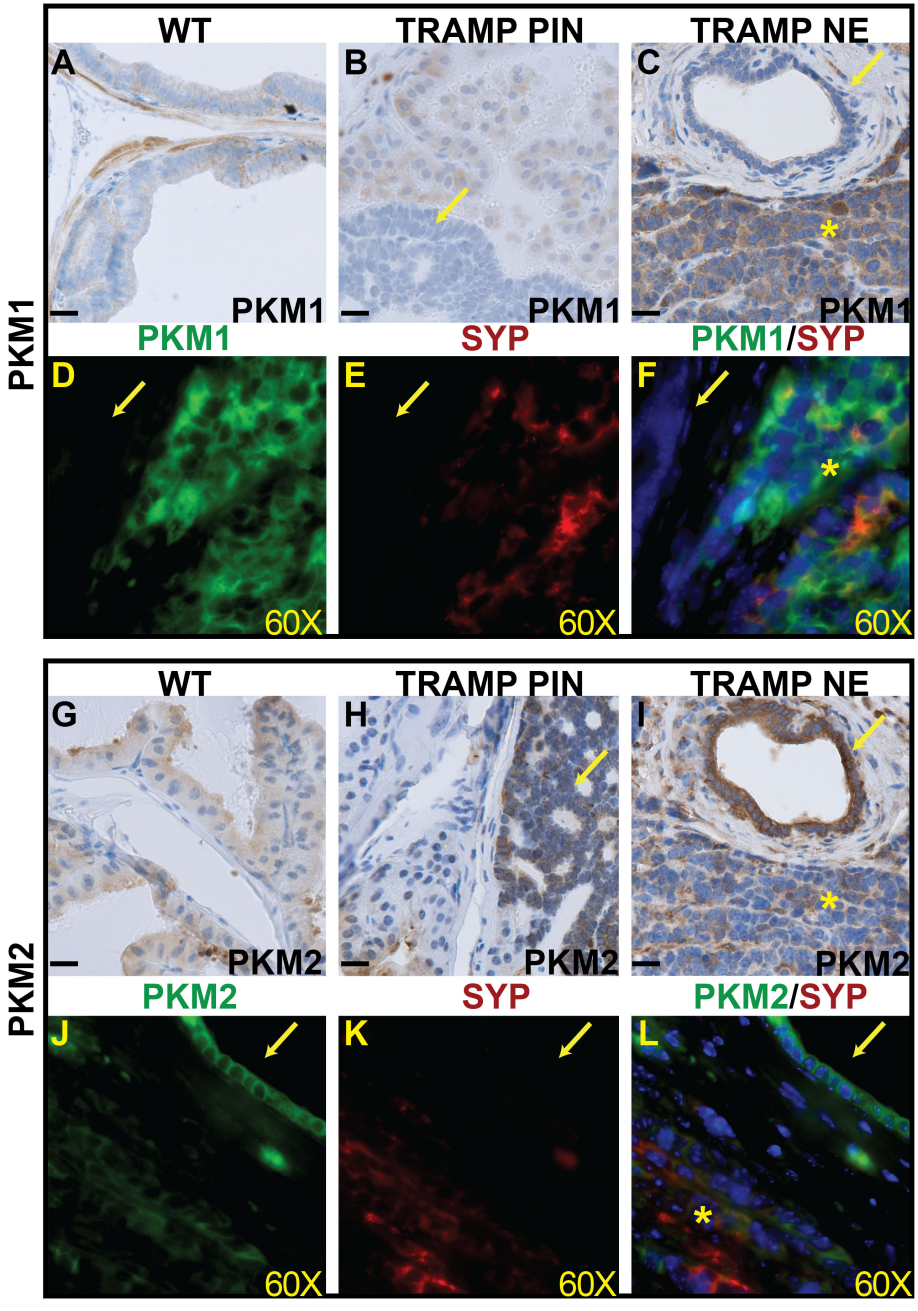
The expression of PKM1 and PKM2 in prostate cancer mouse models. **(A–C)** IHC staining of PKM1 in wild-type prostate **(A)**, TRAMP PIN **(B)** and TRAMP NEPCa tumors **(C)**. **(D–F)** Dual IF staining of PKM1 (**D**, green) with NE marker SYP [**E**, red)] in TRAMP tumor. PKM1 is expressed in NEPCa area but not in adjacent PIN. **(G–I)** IHC staining of PKM2 in wild-type **(G)**, TRAMP PIN **(H)** and TRAMP NEPCa **(I)**. **(J–L)** Dual IF staining of PKM2 (**J**, green) with NE marker SYP [**K**, red)] in TRAMP tumors. PKM2 level is relatively lower in NEPCa area compared with adjacent PIN. The yellow arrows denote prostatic intraepithelial neoplasia (PIN) lesions. Asterisks denote NEPCa. Scale bars for IHC = 20 μM. IF images were taken using 60X lens.

Next, we evaluated the expression of PKM1 and PKM2 in TRAMP tumors. Similar to the previously observed pattern in fully developed 8-week prostates, low to moderate levels of PKM1 expression were noted in the luminal epithelial cells of normal prostates as well as the normal adjacent epithelia of TRAMP tumors (**Figures 2A, B**). However, PKM1 expression was absent in the PIN lesions (**Figures 2B, C**). Interestingly, PKM1 expression was detected in the NEPCa cells (**Figure 2C**). This finding was further corroborated by dual-IF staining. As illustrated in **Figures 2D–F**, PKM1 expression was detected in the NEPCa cells (as indicated by the expression of NEPCa marker SYP), while it was absent in the adjacent PIN lesions.

Robust PKM2 expression was observed in both normal (**Figure 2G**) and transformed prostate epithelial cells (**Figures 2H, I**). It is noteworthy that NEPCa cells exhibited a slightly weaker expression of PKM2 compared to adjacent PIN cells (**Figure 2I**). This finding was further supported by dual IF staining results. As shown in **Figures 2J–L**, PKM2 expression was more pronounced in the PIN lesions compared to the adjacent NEPCa areas, as indicated by the positive staining of SYP.

### 3.2 The expression of PKM1 and PKM2 in human prostatic tissues

We extended our investigation to characterize the expression of PKM1 and PKM2 proteins in human patient samples, including BPH, low-grade AdPCa, high-grade AdPCa, and NEPCa.

**Figure 3:**
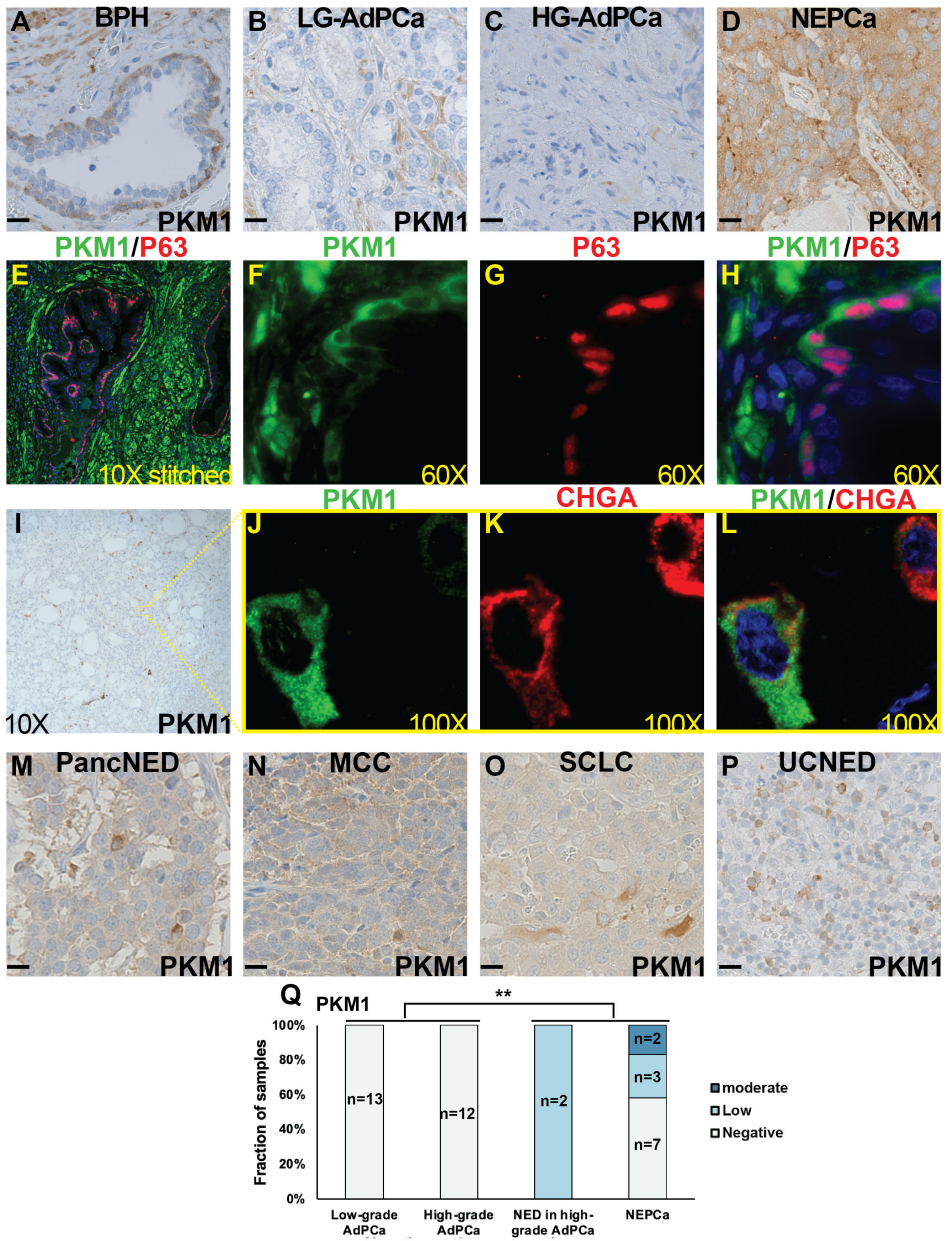
The expression of PKM1 in human tissues. **(A–D)** IHC staining of PKM1 in BPH **(A)**, low-grade (LG) AdPCa **(B)**, high-grade (HG) AdPCa **(C)** and NEPCa **(D)**. **(E–H)** IF staining of PKM1 (**F**, green) and basal epithelial cell marker P63 (**G**, red) in human BPH. PKM1 is expressed in prostate basal epithelial and stromal cells. **(I–L)** Serial sections derived from an AdPCa with NED were used for IHC staining of PKM1 **(I)** or dual IF staining of PKM1 (**J**, green) and NE marker CHGA (**K**, red). PKM1 is co-expressed with CHGA in the scattered NEPCa cells. **(M–P)** IHC staining of PKM1 in pancreatic carcinoma with NED (PancNED) **(M)**, Merkel cell carcinoma (MCC) **(N)**, small cell lung cancer (SCLC) **(O)**, and urothelial carcinoma with NED (UCNED) samples **(P)**. Scale bar for IHC = 20 μM. **(Q)** The quantification of PKM1 IHC staining intensity in human PCa. Chi-Square test **P<0.01, AdPCa (including low-grade and high-grade AdPCa samples) vs NE phenotype (including AdPCa with NE differentiation and NEPCa samples).

Consistent with its expression profile in murine prostate, PKM1 expression in human BPH samples was minimally detected in prostate luminal epithelial cells but exhibited high expression in basal epithelia and stromal cells (**Figure 3A**, n=13). This observation was further confirmed by dual-IF staining. As depicted in **Figures 3E–H**, PKM1 was highly expressed in stromal cells (**Figure 3E**) and basal epithelial cells, as evidenced by the co-expression of basal marker P63.

In human AdPCa (including both low- and high-grade tumors), PKM1 expression was generally sparse (**Figures 3B, C**, n=13 and 12, respectively). However, in the AdPCa with NE differentiation (NED, **Figure 3I**, n=2), PKM1 expression was detected in the scattered NE cells, as indicated by the co-expression of NEPCa marker CHGA (**Figures 3I–L**). Additionally, PKM1 expression was detected in 5 out of 12 human NEPCa specimens (**Figure 3D**). The overall quantification of PKM1 expression in different groups of PCa samples was summarized in a bar plot (**Figure 3Q**). Notably, the expression of PKM1 correlated with NE phenotype (Chi-Square test, p<0.01).

Considering the limited sample number of NEPCa, we expanded our study by examining PKM1 expression in the NE tumors arising from other anatomical sites. We found that PKM1 was expressed in 4 out of 4 pancreatic carcinomas with NED (**Figure 3M**), 2 out of 3 Merkel cell carcinomas (**Figure 3N**), 1 out of 2 small cell lung cancers (**Figure 3O**), and 2 out of 3 urothelial carcinomas with NED (**Figure 3P**). These results suggest a potential role of PKM1 in the pathogenesis of NE tumors.

**Figure 4:**
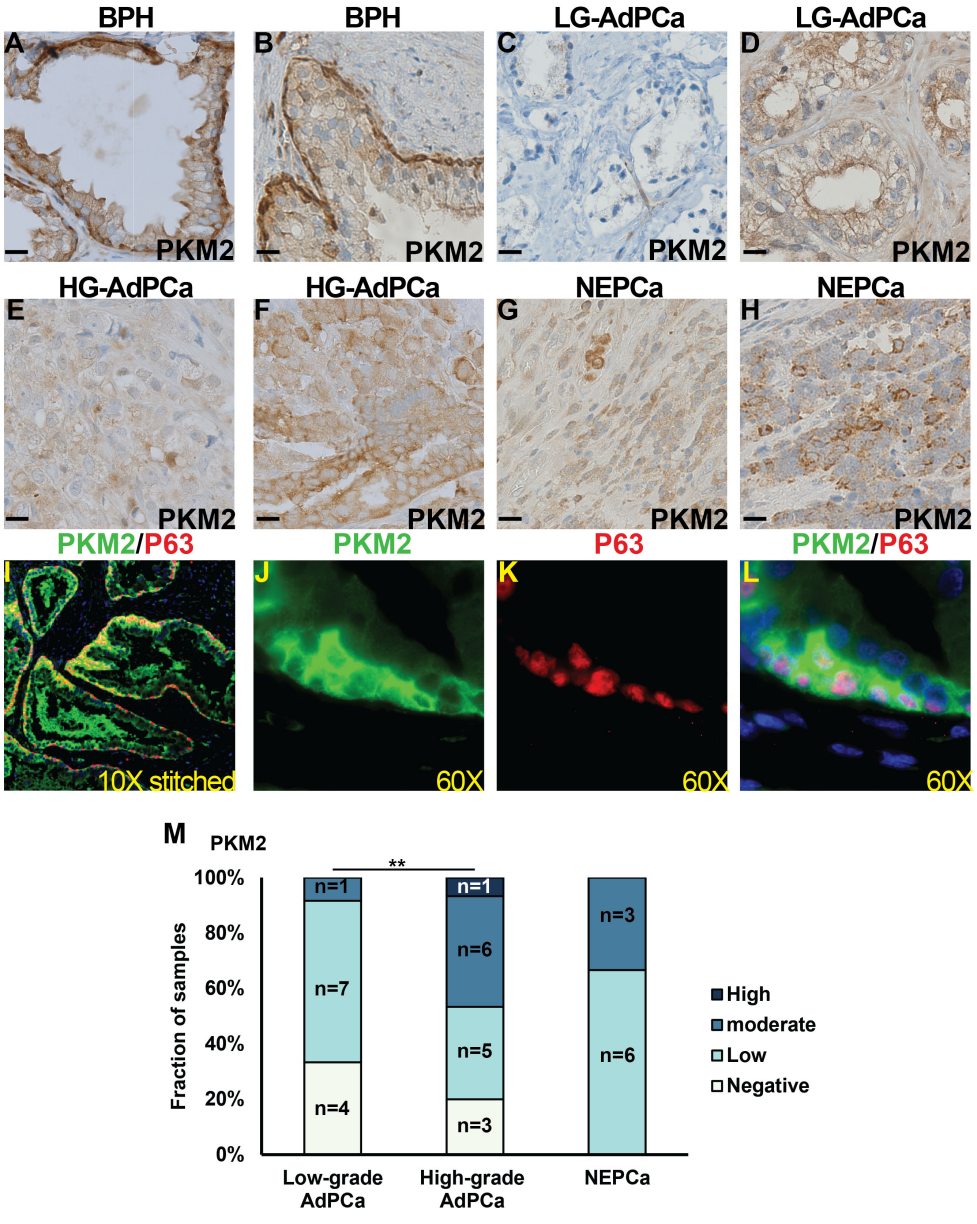
The expression of PKM2 in human prostate specimens. **(A–H)** IHC staining of PKM2 in BPH **(A, B)**, low-grade (LG) AdPCa **(C, D)**, high-grade (HG) AdPCa **(E, F)** and NEPCa **(G, H)**. **(I–L)** IF staining of PKM2 (**B**, green) and basal epithelial cell marker P63 (**C**, red) in human BPH. **(M)** Quantification of PKM2 staining intensity. Scale bar for IHC = 20 μM. Chi-Square test **P<0.01.

On the other hand, the expression of PKM2 was detected in the majority of human prostatic tissues. In the BPH samples, PKM2 expression was prominent in basal epithelial cells and comparatively moderate in luminal epithelial cells (**Figures 4A, B**, n=14). This expression pattern was confirmed by dual IF staining, which demonstrated that PKM2 was highly expressed in basal epithelial cells (as indicated by the co-expression of basal marker P63) with less staining in luminal epithelial cells (**Figures 4I–L**).

Human AdPCa samples exhibited a diverse PKM2 expression pattern, varying from negative to high levels in both low-grade (**Figures 4C, D**, n=12) and high-grade AdPCa (**Figures 4E, F**, n=15). Its expression level was higher in high-grade AdPCa compared to low-grade AdPCa (Chi-Square test, P<0.01). Unlike the decreased PKM2 expression observed in murine NEPCa, PKM2 expression in human NEPCa did not differ significantly from that of AdPCa (**Figures 4G, H**, n=9). The intensity of PKM2 staining in PCa cells, including both AdPCa and NEPCa, was quantified, and its distribution among PCa samples is presented in **Figure 4M**.

### 3.3 The expression of PKM1 and PKM2 in human PCa cell lines

**Figure 5:**
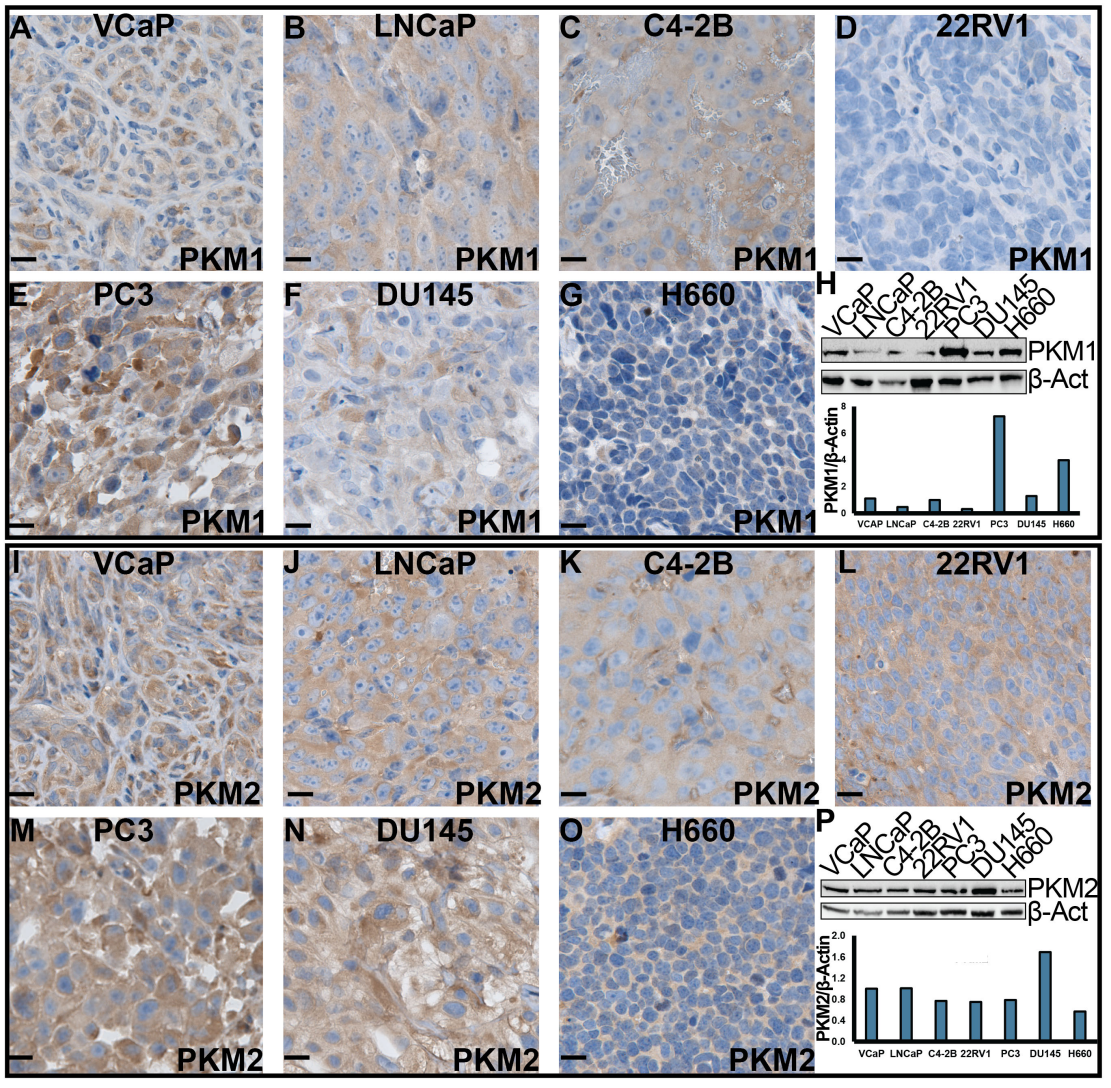
The expression of PKM1 and PKM2 in human PCa cell lines. **(A–G, I–O)** IHC staining of PKM1 and PKM2, respectively, in xenograft tumors derived from human PCa cell lines. Scale bar = 20 μM. **(H, P)** Western blotting analysis of PKM1 **(H)** and PKM2 **(P)** in *in vitro*-cultured human PCa cells.

Furthermore, we assessed the expression of PKM1 and PKM2 in cell lines representing various stages of PCa, including androgen receptor (AR) positive (VCaP, LNCaP, C4-2B and 22RV1), AR negative (PC3 and DU145), and NEPCa (H660) cells. Both *in vitro* cultured cells and cell-derived xenograft tumors were examined. Our IHC staining revealed that the protein expression of PKM1 was lowest in 22RV1- and highest in PC3- derived xenograft tumors (**Figures 5A–G**). This was consistent with the results of Western blot analysis of *in vitro*-cultured cells (**Figure 5H**). NEPCa H660 cells exhibited low to moderate levels of PKM1 expression (**Figures 5G, H**).

Consistent with its expression in human patient samples and murine TRAMP tumors, we observed high protein expression of PKM2 in all the AdPCa cell lines examined (**Figures 5I–N, P**). Its level was relatively lower in NEPCa cell line H660 cells (**Figures 5O, P**).

### 3.4 The expression of ten PKM isoforms in PCa cell lines and patient samples

**Figure 6:**
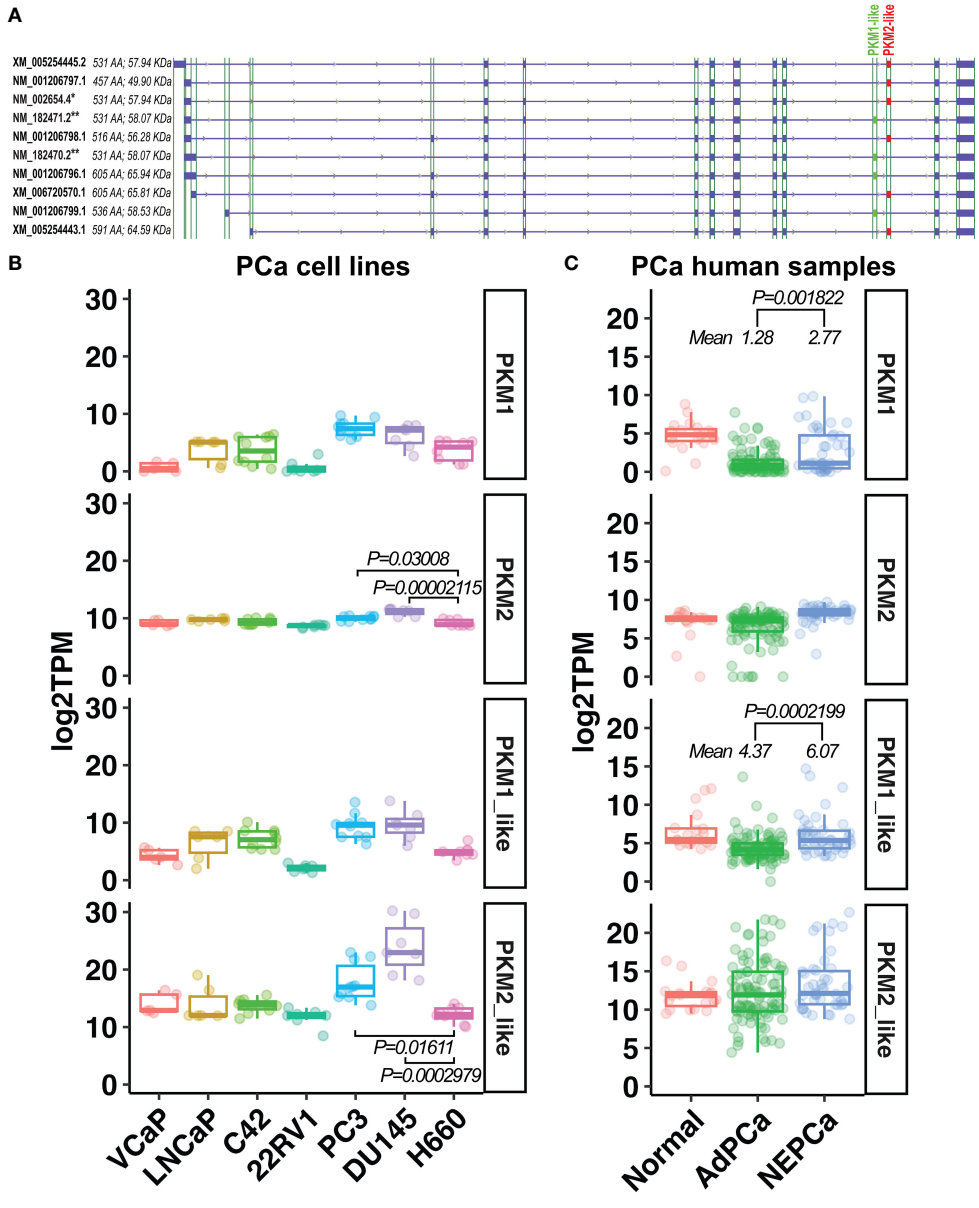
The expression of PKM1-like and PKM2-like isoforms in human PCa. **(A)** Ten PKM isoforms. PKM1-like isoforms are labeled in green and PKM2-like isoforms are in red. ** indicates the previously known PKM1 isoforms, and * indicates the previously known PKM2 isoform. **(B, C)** The levels of the previously known PKM1 and PKM2 as well as the newly classified PKM1-like and PKM2-like transcripts in human PCa cell line and human prostatic tissues.

Using publicly available RNAseq data derived from human PCa cell lines and patient samples, we quantified the transcripts of ten PKM isoforms that have been identified on GRCh38 (release 106). Based on the inclusion of exon 9 or exon 10, these isoforms were classified into “PKM1-like” and “PKM2-like” transcripts, respectively. They include the well-studied PKM1 (NM_182470.2 and NM_182471.2) and PKM2 (NM_002654.4) isoforms, as well as seven under-studied isoforms (**Figure 6A**). The expression levels of these isoforms were summarized in **Supplementary Figure 1**. Notably, the previously mentioned PKM1 and PKM2 antibodies, targeting exon 9 or 10, detect the protein products of PKM1-like or PKM2-like transcripts.

As shown in **Figures 6B, C**, there was a wide variation in the expression of PKM1 transcripts among PCa cell lines and human prostatic tissues. Its level was higher in human NEPCa specimens compared with AdPCa samples (**Figure 6C**).

Overall, the levels of PKM2-like transcripts were higher than that of PKM1-like transcripts in both PCa cell lines and human PCa specimens (**Figures 6B, C**). Among the PKM2-like isoforms, NM_002654.4 transcript was the most abundant one expressed in PCa cell lines and human prostatic tissues (**Supplementary Figure 1**), and its level was lower in NEPCa cell line H660 compared to the aggressive PCa cell lines PC3 and DU145 (**Figure 6B**, t-test, p<0.05).

Additionally, we analyzed the prostatic expression of PKL/R, the other two PK isoforms (41). We found that the levels of PKL/R transcripts were generally low in human PCa cell lines and human PCa samples. However, these transcripts were detected in a subset of human NEPCa samples (**Supplementary Figure 2**). This finding is consistent with a recent study indicating that PKL/R expression is increased in castrate-resistant PCa, and the upregulated PKL/R, along with elevated MYCN, may drive metabolic reprogramming during NE differentiation (46).

### 3.5 The stoichiometry of PKM2

**Figure 7:**
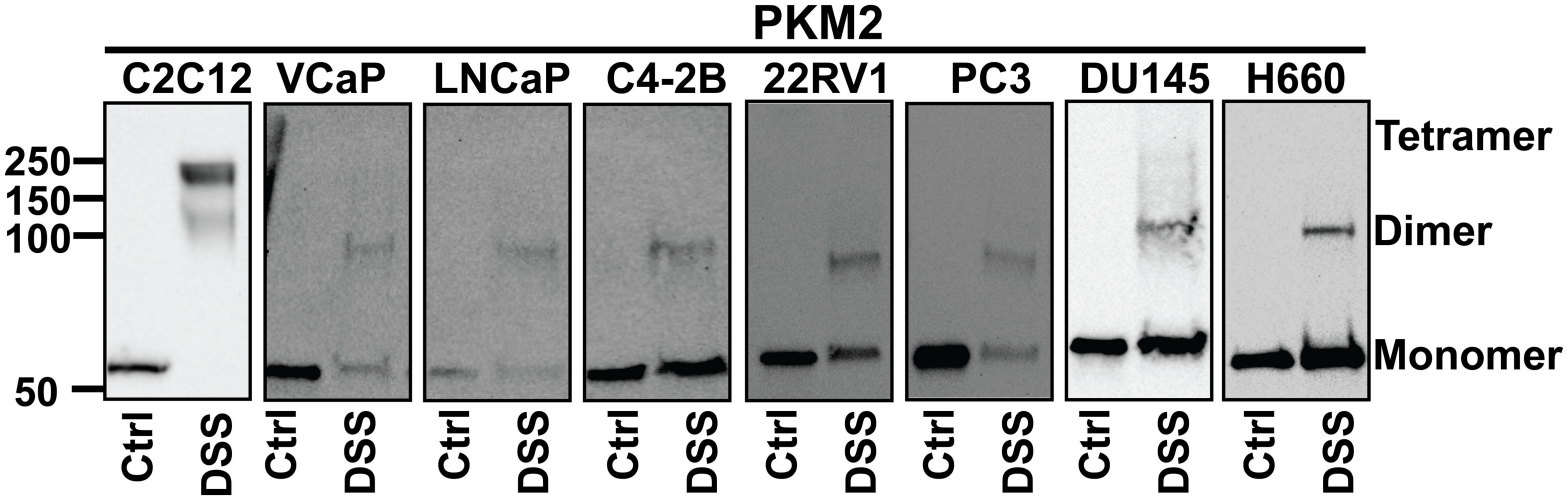
The stoichiometry of PKM2. Cells were treated with disuccinimidyl suberate (DSS) reagent to form intramolecular crosslinks and Western blot analysis was conducted to examine the subunit association of PKM2. PKM2 primarily forms monomers and dimers but not tetramers in PCa cells. C2C12 cells were used as a positive control for the detection of PKM2 tetramers.

To study the stoichiometry of PKM in PCa, we conducted a cross-linking reaction. While PKM1 was hardly detected after cross-linking through Western blotting due to the low expression level, the multi-unit formation of PKM2 was visible. C2C12 myoblast cells were used as a positive control for the detection of PKM2 tetramers (**Figure 7**). We found that in PCa cells, PKM2 primarily formed inactive monomers/dimers rather than active tetramers (**Figure 7**).

## 4 Discussion

In this study, we examined the expression profiles of PKM1 and PKM2 at both protein and mRNA levels in the context of PCa, utilizing samples from human patients, mouse models, and human PCa cell lines. Our findings reveal a heterogeneous expression pattern of PKM1 across different PCa stages. Particularly noteworthy is the observation of elevated PKM1 expression in a subset of NEPCa. This finding is in line with existing literature, which indicates that PKM1 is expressed in NE tumors derived from other organs, such as small cell lung cancer (47, 48), suggesting a distinct metabolic status of NE tumors compared to their adenocarcinoma counterparts. Additionally, our findings suggest that oxidative phosphorylation may play a significant role in ATP production in NE tumors.

In contrast, PKM2 demonstrates abundant expression in PCa tissues across various stages. Interestingly, its protein expression is lower in murine NEPCa cells compared to AdPCa. This observation extends to PCa cell lines but not consistently in human PCa specimens, likely due to the intricate genetic profiles of human specimens and the limited availability of NEPCa samples.

Collectively, this study represents the first investigation into the expression of PKM isoforms in NEPCa, laying the groundwork for further exploration into its energy metabolism. Accumulating evidence suggests that NEPCa arises from AdPCa following androgen deprivation therapy. It would be intriguing to study whether the expression of PKM isoforms in PCa is regulated by AR signaling.

Although the antibodies targeting exon 9 or 10 can detect the PKM1- and PKM2- like proteins, respectively, they lack the ability to distinguish between individual isoforms or facilitate comparisons of their expression levels due to variations in binding affinity between antibody-antigen pairs. However, this limitation was mitigated by our RNAseq analysis, which elucidated the expression of ten PKM isoforms in prostatic tissues. Importantly, the analysis revealed that PKM2 is the predominant isoform expressed in PCa cells.

While PKM1 forms a constitutively active tetramer, PKM2’s stoichiometry is regulated by allosteric regulators such as serine and fructose 1, 6-bisphosphate (FBP), as well as post-translational modifications, leading to the formation of less-active monomers and dimers (22, 30, 31). Our data indicate that PKM2, the isoform predominantly expressed in PCa, adopts a less-active stoichiometry. In these cells, the conversion of PEP to pyruvate is slowed down, potentially resulting in the accumulation of glycolytic intermediates, which can be diverted into anabolic pathways to fuel cell proliferation (22).This observation is consistent with literature suggesting that PKM2 promotes the Warburg effect in PCa (34, 36).

In summary, this study characterized the expression of PKM1 and PKM2 in prostatic tissues including developing, benign, and cancerous prostate. These findings enhance our understanding of the metabolic alterations in aggressive PCa. However, due to the scarcity of NEPCa specimens, further research is warranted to fully elucidate the metabolic profile of this aggressive PCa subtype.

## Data availability statement

The public data presented in this study were accessed through GEO (https://www.ncbi.nlm.nih.gov/geo/), dbGaP (https://www.ncbi.nlm.nih.gov/gap/) and GDC Data Portal (https://portal.gdc.cancer.gov/). All codes necessary to reproduce figures and statistic results included in this manuscript are available in GitHub (https://github.com/schoo7/PKMisoforms).

## Ethics statement

Ethical approval was not required for the studies on humans in accordance with the local legislation and institutional requirements because only commercially available established cell lines were used. The animal study was approved by ACUC and IRB of LSU Health-Shreveport. The study was conducted in accordance with the local legislation and institutional requirements.

## Author contributions

LL: Writing – original draft, Visualization, Investigation, Writing – review & editing, Formal analysis, Data curation. SC: Writing – review & editing, Software, Formal analysis, Data curation. YY: Writing – review & editing, Formal analysis, Data curation. YS: Writing – review & editing. NH: Writing – review & editing. DP: Writing – review & editing. XG: Writing – review & editing. XY: Writing – review & editing, Supervision, Resources, Project administration, Funding acquisition.

## Supplementary material

Supplementary Figure 1 The mRNA expression of PKM isoforms in PCa cell lines **(A)** and human prostate specimen **(B)**.

Supplementary Figure 2 The mRNA expression of PKLR in PCa cell lines **(A)** and human prostate specimen **(B)**.
