# Supplementary figures and images for: The expression of PKM1 and PKM2 in developing, benign, and cancerous prostatic tissues
Source: Front Oncol. 2024 Apr 12;14:1392085. doi: 10.3389/fonc.2024.1392085 (PMC11045992; doi:10.3389/fonc.2024.1392085)

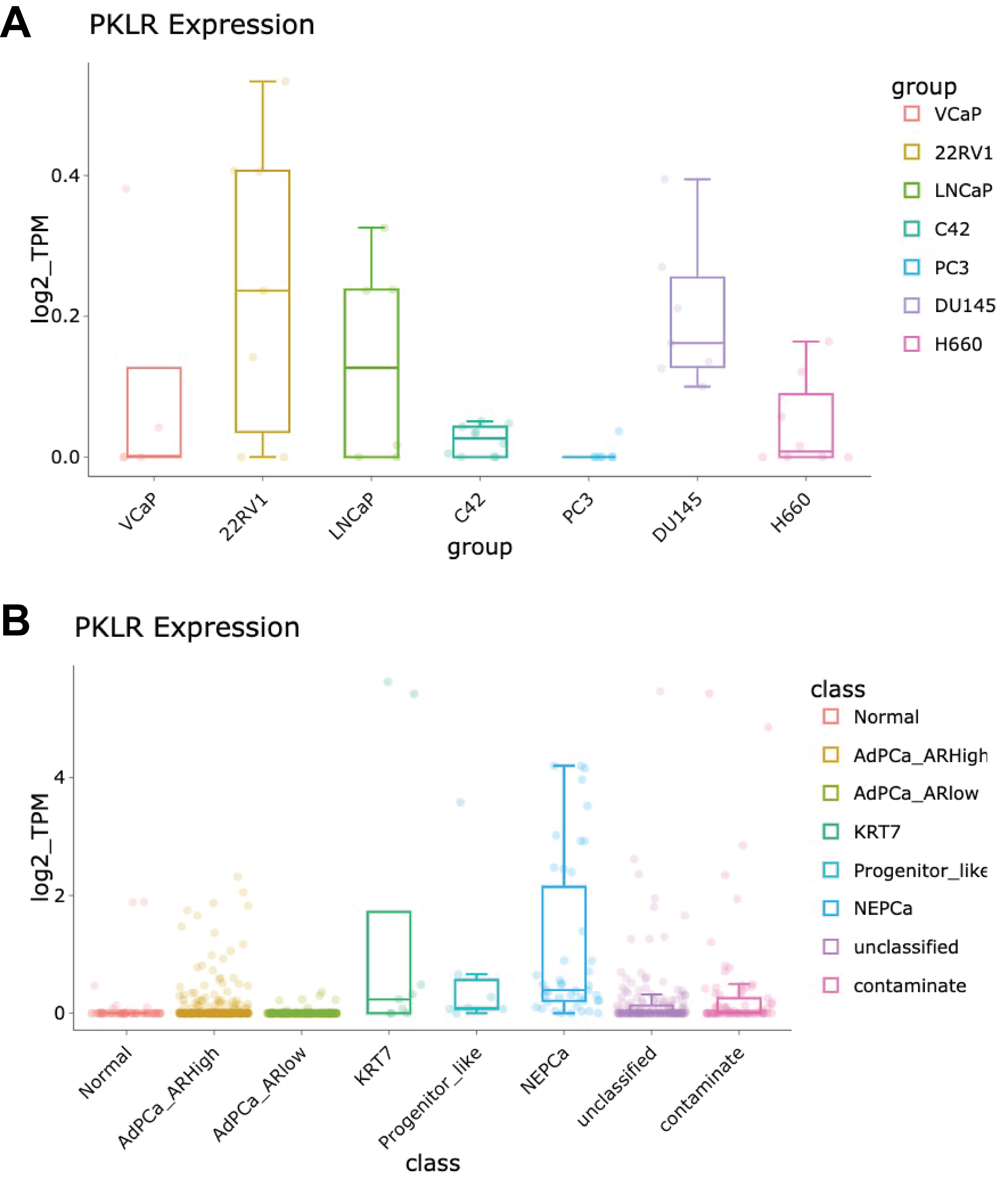

Supplement: Supplementary Figure 2 — The mRNA expression of PKLR in PCa cell lines (A) and human prostate specimen (B). [file Image_2.tif]
